# Characteristic of the Nanoparticles Formed on the Carbon Steel Surface Contacting with 3d-Metal Water Salt Solutions in the Open-Air System

**DOI:** 10.1186/s11671-016-1267-2

**Published:** 2016-02-04

**Authors:** O. M. Lavrynenko, O. Yu Pavlenko, Yu S. Shchukin

**Affiliations:** F.D. Ovcharenko Institute of Bio-Colloid Chemistry of NAS of Ukraine, Vernadsky Ave, 42, 03142 Kyiv, Ukraine

**Keywords:** The rotation-corrosion dispergation method, Mixed layered double hydroxides, Thermal behavior, Spinel ferrites, 61.46.-w, 81.07.-b, 81.16.-c

## Abstract

The contact of a steel electrode with water dispersion medium in an open-air system leads to the development of various polymorphic iron oxides and oxyhydroxides on the steel surface. Whereas the usage of distilled water causes the obtaining of Fe(II)-Fe(III) layered double hydroxides (green rust) as a primary mineral phase, but in the presence of inorganic 3d-metal water salt solutions, mixed layered double hydroxides (LDHs) together with non-stoichiometric spinel ferrite nanoparticles are formed on the steel surface. Mixed LDHs keep stability against further oxidation and complicate the obtaining of spinel ferrite nanoparticles. Thermal treatment of mixed LDHs among other mineral phases formed via the rotation-corrosion dispergation process at certain temperatures permits to obtain homogenous nanoparticles of spinel ferrites as well as maghemite or hematite doped by 3d-metal cations.

## Background

Nowadays, iron–oxygen-containing nanoparticles due to their high catalytic and magnetic properties are widely used as precursor species for the creation of various materials for technical and bio-medical application [1, 2]. The presence of 3d-metal cations such as copper, cobalt, nickel, and zinc in the crystal lattice of iron oxides and oxyhydroxides significantly changes their physical–chemical properties and may be important for enhancing the quality of the obtained materials as well as for imparting new properties to the existing materials [3, 4]. Typical methods for obtaining iron-oxygen high-disperse particles are well known and described in numerous reviews [5, 6]. But from our point of view among the diversity of the existing methods, the formation of the iron-bearing nanoparticles on the iron or steel surface has a distinct advantage because the presence of an anionic part (chlorides, sulfates, nitrates, etc.) in the reaction area is insignificant.

Recently, we have proposed an alternative method for the formation of various iron-oxygen mineral nanoparticles, including spinel ferrites, on the surface of the steel electrode contacting with air and water medium that was called the rotation-corrosion dispergation (the RCD) [7]. The kinetic study of the processes of the phase formation on the steel surface contacting with distilled water showed the appearance of Fe(II)-Fe(III) layered double hydroxides (LDHs) or green rust as the primary (first nucleus) phase within 1–3 h. The presence of ferrous cations in the hydroxide layers makes the green rust structure unstable to further dissolution or oxidation. While, the presence of 3d-metal inorganic salts in the dispersion medium contacting with the iron or steel surface provides the nucleation of mixed Fe–3d-metal layered double or triple hydroxides, whose physical–chemical properties significantly differ from the properties of the chemically pure Fe(II)-Fe(III) LDHs. It is known that the typical mixed LDHs are remarkable for their full oxidation of ferrous iron in a crystal lattice and hence they lose reductive properties as well as capability for phase transformation under standard conditions. But not only iron changes its oxidation level in the LDH lattice, because the same properties are typical for other cations like copper or cobalt [8]. As a result, the appearance of the mixed LDH structures on the steel surface can substantively complicate the obtaining of spinel ferrite nanoparticles on the steel surface when the RCD method is applied.

The purpose of our work is to study the role of anionic composition of the water solutions contacting with iron or steel surface under the rotation–corrosion dispergation in the formation of mixed layered double hydroxides and their further transformation in polymorphic iron oxyhydroxides and oxides.

## Methods

The formation of nanosized iron-oxygen mineral particles on the carbon steel surface was performed using a rotating disk electrode that periodically contacted with 3d-metal water salt solutions and the air. The disk electrode was made of finished steel (St3) that has the following compositions in percentage: С—0.14–0.22; Si—0.05–0.15; Mn—0.4–0.65; Cr—0.3; Ni—0.3; P—0.04; S—0.05; and N—0.01. The steel surface was exposed to mechanical treatment and further activation using concentrated sulfuric acid. The activated disk electrode was repeatedly rinsed in distilled water, and onwards, it was placed into the cell filled with the 3d-metal water salt solutions. We have chosen the following eight solutions: cobalt, zinc, nickel, and copper chlorides and sulfates as the dispersion medium. The concentration of the metal cations in the water solutions equaled 100 mg/l, and the pH value was set as 6.5. The formation of disperse nanostructures on the steel surface lasted 24 h at around *T* = 20 °C. The temperature conditions were set using TS-1/80-SPU thermostat. Afterwards, the disk electrode was dried in the air atmosphere and the surface nanostructures were exposed to a complex physical–chemical investigation including an X-ray diffraction (XRD) method, thermal analytical measurements (TG/DTG, DTA), and scanning electron microscopy (SEM). The phase composition of the surface nanostructures, their phase transformation and thermal behavior, morphology, and coherent scattering region (CSR) were determined as the objects of the study.

The phase composition of the surface structures was determined using the computer-aided X-ray diffractometer (DRON-UM1) equipped with two Soller’s slits and filtered radiation of cobalt anode Co*K*_α_. The rate of recording was set at 1°/min, and the interfacial Woolf-Bragg’s angle made up 80–90°. The coherent scattering region characterizing the size of primary particles or crystallites was calculated according to the standard Debye-Scherrer’s formula.

For the calculation of CSR, we chose the peaks of (311) for the spinel (JCPDS file No19-0629), (020) for the lepidocrocite (JCPDS file No08-0098), (110) for the goethite (JCPDS file No17-536), (003) for green rust I (JCPDS file No40-0127), and (003) for green rust II (JCPDS file No41-0014). The reason for this choice is the presence of these reflexes in all samples and the fact that each reflex is not overlapped by the reflex of another iron–oxygen-containing phase. Apart from the aforementioned, the reflexes at large scattering angles have a small intensity on some XRD patterns, and they also may be less suitable for lattice parameters calculation.

A simultaneous study of thermogravimetric and differential thermal properties (TG-DTA) of the surface structures was performed in a static air atmosphere by the derivatigraph Q-1500D (Hungary). The record was made using computer data registration. The parameters of the pattern recording were the following: the samples 35.2–102.6 mg were heated at the rate of 10 °С/min from 20 to 1000 °С; the sensitivity was 20 mg; and TG—500, DTG—500, and DTA—250. The samples were placed into a corundum crucible and covered by a quartz beaker to create an equal temperature field. A SEM using JOEL-6700 microscope equipped with an energy-dispersive and cathode-luminescence attachment was chosen as the main method of the research. The mass ratio of iron to the second 3d-metal in the samples was determined using an X-ray fluorescence spectroscopy (XRFS) carried out in the automatic spectrometer «ElvaX» equipped with a titanium anode.

## Results and Discussion

At the first stage of the process, we deliberately formed the layer of the primary mineral phase on the steel surface via contact of the activated steel electrode with distilled water at *T* = 20 °C. Free access of the air into the reaction area and the absence of other anionic species in the dispersion medium led to the formation of hydroxycarbonate Fe(II)-Fe(III) LDH or green rust (GR)(CO_3_^2–^). Development of such a structure on the steel surface contacting with water dispersion medium in the temperature range from 3 to 70 °C was in detail described in our previous work [9].

The various micellar ferric and ferrous species appear in the reaction area (spreading for ~400 μm from the steel surface) at the average pH value in the range from 7.0 to 9.5 where the numerous products of electrochemical processes and their hydrated and/or oxidized forms (Fe(OH)_3_, Fe^2+^, FeOH^+^, Fe(OH)_2_, and OH^−^) simultaneously interact with each other [7]. Protons, oxygen, and oxygen-containing carbon species belong to other active components in the system. The latter exists in the reaction area as carbon dioxide or carbonic acid as well as the anions –НСО_3_^−^, СО_3_^2−^, and they can interact with dissolved ferrous species and form various aquacomplexes, e.g., FeHCO_3_^+^, Fe(HCO_3_)_2_, Fe(CO_3_)_2_^2−^ [10].

General reaction of GR(CO_3_^2−^) formation may be written as

$$ 6\mathrm{F}\mathrm{e}{\left(\mathrm{O}\mathrm{H}\right)}_{2\ \mathrm{solid}} + 0.5{\mathrm{O}}_{2\ \mathrm{gas}} + \mathrm{C}{{\mathrm{O}}_2}_{\mathrm{gas}} + 3{\mathrm{H}}_2\mathrm{O}\ \to\ \mathrm{F}{{\mathrm{e}}^{\mathrm{II}}}_4\mathrm{F}{{\mathrm{e}}^{\mathrm{II}\mathrm{I}}}_2{\left(\mathrm{O}\mathrm{H}\right)}_{12}\mathrm{C}{\mathrm{O}}_3\cdot 3{\mathrm{H}}_2{\mathrm{O}}_{\mathrm{solid}} $$

Obviously, the presence of ferrous iron in the green rust lattice causes its oxidation under open-air conditions. The further development of hydroxycarbonate green rust on the carbon steel surface contacting with distilled water is carried out via its solid-state oxidation into Fe(III)–green rust or lepidocrocite [11]. But lepidocrocite is a metastable phase with respect to goethite [12], and the presence of a small amount of Fe^2+^ and CO_2_ catalyzes such polymorphous phase transformation via a dissolution–re-precipitation route [13], whereas a large access of Fe^2+^ to the lepidocrocite surface causes re-precipitation of spherically shaped magnetite particles [12].

**Fig. 1:**
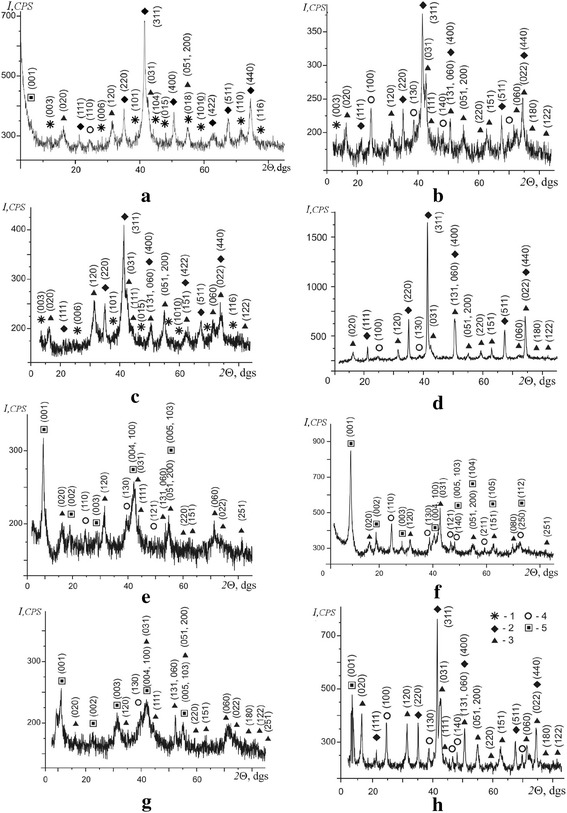
XRD patterns of the phases formed on the steel surface. The concentration of 3d-metal cations equals 100 mg/dm^3^ and pH = 6.5. **a** CoCl_2_. **b** NiCl_2_. **c** ZnCl_2_. **d** CuCl_2_. **e** CoSO_4_. **f** NiSO_4_. **g** ZnSO_4_. **h** CuSO_4_. Numbers correspond to the following phases: 1—green rust I, 2—Fe_3_O_4_, 3—γ-FeOOH, 4—α-FeOOH, 5—green rust II

The presence of 3d-metal cations (Co, Ni, Zn, and Cu) in the water dispersion medium contacting with steel leads to the formation of several mineral phases on its surface. According to the X-ray diffraction data (Fig. 1), the phase composition of disperse sediments contains iron oxyhydroxides (lepidocrocite γ-FeOOH and goethite α-FeOOH), spinel ferrites (magnetite containing various 3d-metal cations in its crystal lattice), as well as mixed layered double hydroxides correspond to the first or second type of the green rust’s lattice: hydrotalcite-like green rust I (hydroxycarbonate or hydroxychloride) and hydrohonessite-like green rust II (hydroxysulfate), respectively.

The mineral association of *lepidocrocite*–*pinel ferrite* dominates in cobalt- and zinc-bearing systems. Such association is expanded with goethite as a third member in nickel- and copper-bearing systems. Other features of the mineral composition of the surface nanostructures are the following:

1. The formation of spinel ferrite particles takes place when the steel surface is contacting with chloride-containing solutions with one exception of CuCl_2_ system.
2. Green rust I displays only weak XRD reflexes, but green rust II peaks are well developed.
3. Weak crystallinity and small particle size are typical for the mineral phases formed in all cobalt- and zinc-containing systems and for the nickel chloride one. But the well crystalline spinel ferrite phases are formed in both copper-containing systems and, likely, in such systems, the LDH part may be insignificant.

After the growth of the GR(CO_3_^2−^) on the steel electrode and placing it into the cell filled with various inorganic salt solutions, the hydroxycarbonate LDH becomes an ion exchange substance. The fundamental research of the equilibrium constants of the exchange processes calculated for various LDH structures points to an anion competition in the green rust interlayer space and shows a higher selectivity of divalent anions in comparison with the monovalent ones [14]. In the context of our study, the comparative list of the green rust stability, depending on its anion composition, is as follows: Cl^−^ < CO_3_^2−^ < SO_4_^2−^. But according to [15], the stability of green rust structure against oxidation increases in the row Cl^−^ < SO_4_^2−^ < CO_3_^2−^. Also, carbonate anions may be deintercalated from the LDH lattice when a large access of other anions (Cl^−^) is supplied [16]. The nature of incorporated anion (nitrate, chloride, and carbonate) determines the phase composition of the final substance, its basal spacing, points of zero-charge as well as thermal behavior, optical properties, and catalytic activity as it was illustrated by the example of mixed Fe-Zn containing the LDHs, obtained by the co-precipitation method [17].

At the same time, such cations as Cu^2+^, Ni^2+^, Zn^2+^, Cd^2+^, Co^2+^, and Mg^2+^ may isomorphically substitute Fe(II) during green rust formation [18]. The relative stability of mixed LDHs is higher in comparison with the individual hydroxides: it strongly depends on the kind of included divalent cations and increases in the row: Co^2+^ ~ Ni^2+^ < Zn^2+^ and, generally, it is higher in comparison with the individual hydroxides [19]. At that, the anion composition (type) of the green rust lattice does not influence the capability to exchange processes. Also, as it was shown in the example with nickel-bearing systems, Ni^2+^ can substitute Fe(II) in both green rust I (GRI) [20] and green rust II (GRII) [21].

**Fig. 2:**
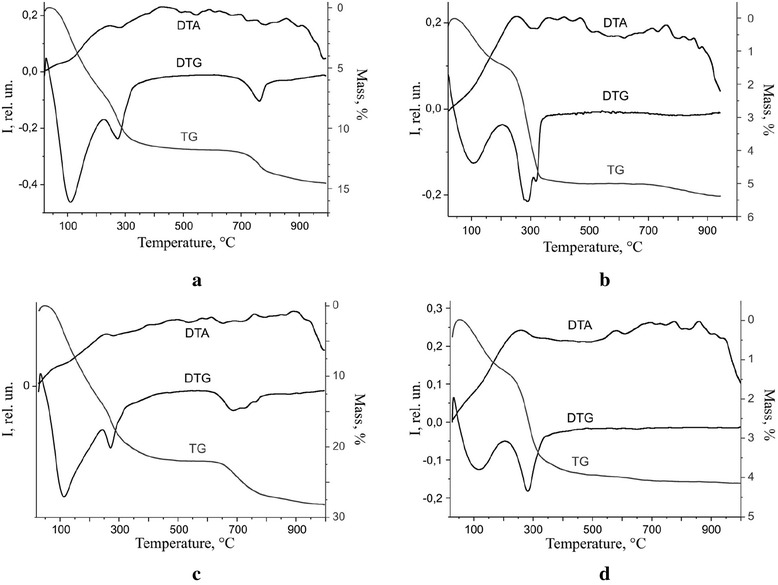
The TG-DTA curves for the phases formed on the steel surface. The 3d-metal water salt solutions contacting with the steel surface: **a** CoSO_4_, **b** CuSO_4_, **c** NiSO_4_, **d** NiCl_2_

Given that the GRI structure is less stable in comparison with GRII, in our case, mixed LDHs, related to the first type (GR(Cl^−^) or GR(CO_3_^2−^)), quickly transformed into ferric oxyhydroxides or spinel ferrites under the RCD conditions as we can see in the XRD patterns (Fig. 1a–d), though mixed LDHs related to the second type (GR(SO_4_^2−^)) remain stable within a relatively long term (Fig. 1e–g) at sufficiently high temperatures (Fig. 2a, c).

The chemical composition of a dispersion medium not only determines the type of green rust or mixed LDH lattice but also provides the crystallization of one or another polymorphous modification of ferric oxyhydroxides. The formation of goethite α-FeOOH may be enhanced in the presence of SO_4_^2−^ anions [22], whereas the role of carbon dioxide in the formation of goethite versus lepidocrocite γ-FeOOH in solutions was pointed in [13]. The poorly crystalline γ-FeOOH was formed in the presence of Zn cations in water solutions, but only a trace quantity of α-FeOOH appeared in the zinc substituted samples [23]. Copper inhibits the crystal growth of both goethite and lepidocrocite, whereas nickel is included into the goethite lattice without destructing its crystal structure [24, 25]. Moreover, the presence of zinc and copper cations protects the lepidocrocite particles against dissolution in ferrous sulfate medium, but nickel ions do not affect the morphology and crystallinity of γ-FeOOH [26]. Ferrous cations are usually adsorbed on the lepidocrocite surface in a water medium and may be substituted for cobalt and nickel cations to form surface-modified iron oxides [15]. The analysis of our system confirms that only γ-FeOOH appeared on the steel surface contacting with ZnSO_4_ and ZnCl_2_ water solutions, and at the same time, both γ-FeOOH and α-FeOOH were present in the phase composition of the sediments when the steel surface was contacting with cobalt-, nickel-, and copper-containing systems. At that, the relative quantity of goethite part is less in chloride-containing solutions in comparison with sulfate-containing medium.

Generally, the particle size of the spinel ferrites, obtained when a few various LDH species were mixed, was smaller in comparison with the size of ferrites formed from individual LDH precursors [27]. The calculation of the lattice parameters and CSR of iron oxyhydroxide and spinel ferrite is summarized in Table 1. The lattice parameters and CSR of iron oxyhydroxide and spinel ferrite depend on the chemical composition of the dispersion medium. So, the particle size of spinel ferrites ranges from 16 to 28 nm, lepidocrocite size varies from 12 to 20 nm, and goethite from 13 to 18 nm, respectively. Lattice parameters (*a*, *c*) and CSR (*d*) of mixed hydroxysulfate LDHs (green rust II) are shown in Table 2. The interplanar distance between two successive Fe^2+^ and Fe^3+^ planes in typical hydroxysulphate green rust equals to 1.095–1.105 nm. But its oxidized form (exGRs-Fe(III), ex-green rust II or Fe(III)–green rust II) is characterized by insignificantly smaller interplanar distance 1.090 nm and indicates some relics of lamellar elements that remain in the structure of oxidized GR [28]. The decrease in the interplanar distance was explained by the oxidation of ferrous iron and related to the difference in the ionic radii of Fe^3+^ (0.0645 nm) and Fe^2+^ (0.078 nm) [11]. Also, according to the XRD data, we suppose that the iron in the crystal lattice of mixed LDHs obtained on the steel surface under the RCD conditions is only partially oxidized and it is present in both ferric and ferrous forms.

**Table 1:** The lattice parameters and CSR of the lepidocrocite, goethite, and spinel ferrite phases

Water salt solutions	Mineral phases formed on the steel surface contacting with water salt solutions					
	γ-FeOOH		α-FeOOH		MeFe_2_O_4_	
	The lattice parameters, nm	The particle size, nm	The lattice parameters, nm	The particle size, nm	The lattice parameters, nm	The particle size, nm
CoCl_2_	*a—*0.3865 *b*—1.2400 *c*—0.3061 V—0.1467	17.6	*a*—0.4611 *b*—1.0178 *c*—0.2998 V—0.1407	16.6	*a*—0.8293 V—0.5703	18.7
NiCl_2_	*a*—0.3855 *b*—1.2681 *c*—0.3024 V—0.1478	18.6	*a*—0.4648 *b*—0.9975 *c*—0.3029 V—0.1404	18.4	*a*—0.8397 V—0.5920	16.8
ZnCl_2_	*a*—0.3876 *b*—1.2638 *c*—0.3067 V—0.1502	21.6	The phase is absent		*a*—0.8395 V—0.5917	16.4
CuCl_2_	*a*—0.3871 *b*—1.2601 *c*—0.3054 V—0.1490	12.4	*a*—0.4668 *b*—0.9740 *c*—0.3043 *V*—0.1384	18.32	*a*—0.8394 V—0.5914	18.5
CoSO_4_	*a*—0.3866 *b*—1.2501 *c*—0.3059 V—0.1478	20.1	a—0.4629 b—1.0854 c—0.2991 V—0.1503	14.8	*a*—0.8299 V—0.5716	15.9
NiSO_4_	*a*—0.3876 *b*—1.2546 *c*—0.3047 V—0.1482	13.7	*a*—0.4630 *b*—0.9969 *c*—0.3016 V—0.1392	14.53	*a*—0.8370 V—0.5864	24.8
ZnSO_4_	*a*—0.3858 *b*—1.2692 *c*—0.3062 V—0.1499	21.7	*a*—0.4626 *b*—1.0106 *c*—0.3013 V—0.1408	16.2	*a*—0.8404 V—0.5936	19.1
CuSO_4_	*a*—0.3875 *b*—1.2565 *c*—0.3058 V—0.1489	13.3	*a*—0.4504 *b*—1.1904 *c*—0.2993 V—0.1605	13.5	*a*—0.8377 V—0.5878	24.7

**Table 2:** The lattice parameters and CSR of the mixed LDH phases formed on the steel surface

Water salt solutions	The lattice parameters, nm	The particle size, nm
CoSO_4_	*a* = 0.3161, *c* = 1.0948	7.9
ZnSO_4_	*a* = 0.3172, *c* = 1.1013	16.2
NiSO_4_	*a* = 0.3219, *c* = 1.1021	15.6

The distribution (mass. %) of iron and the second 3d-metal (Me) in the mineral sediments was studied using X-ray fluorescence spectroscopy. The obtained data are shown in Table 3. The results point to the fact that the ratio of Fe to Me depends on the chemical composition of water solutions and it changes from 94:6 to 79.5:19.5. Its minimum corresponds to both copper-containing systems and maximum relates to zinc- and nickel-containing systems. But the dependence of the Fe to Me ratio on the anion composition of the dispersion medium is the most demonstrative in cobalt-containing systems.

**Table 3:** Distribution of 3*d*-metals in the surface sediments

The cations	The anions			
	SO_4_^2−^		Cl^−^	
	Fe, mass. %	Me, mass. %	Fe, mass. %	Me, mass. %
Cu^2+^	94.0	6.0	91.1	8.9
Co^2+^	92.2	7.8	84.6	15.4
Ni^2+^	82.0	18.0	87.3	12.7
Zn^2+^	84.1	15.9	79.5	20.5

We reach the conclusion that the 3d-metal cations may be associated not only with spinel ferrite phases but with mixed layered double hydroxides as well as ferric oxyhydroxides. In addition, both the spinel ferrites and mixed LDHs belong to the non-stoichiometric mineral phases and the first can be distinguished as the magnetite doped by 3d-metal cations.

The structural stability of the surface mineral phases was examined by simultaneous TG/DTA investigation. The TG and DTA curves are present in Fig. 2. The analysis of DTG curves (Fig. 2) shows the appearance of the first exoeffect related to the removal of the adsorbed water in the temperature range from 106 to 113 °C. The phase transformation of magnetite into maghemite takes place in the temperature range from 200 to 240 °C. Lepidocrocite dehydroxylation process is accompanied by the appearance of the exoeffect in the temperature range from 272 to 281 °C. In addition, an endoeffect at temperature 317 °C and small endoeffect at 308 °C are fixed in CuSO_4_ system (Fig. 2b). Obviously, they correlate with goethite dehydroxylation and its transformation into an iron oxide phase. The phase transformation of maghemite into hematite is noted in the temperature range from 324 to 340 °C. At the same time, an additional endoeffect at temperature 761 °C is fixed in the CoSO_4_ system (Fig. 2a) and a wide (multi)graded endoeffect in the temperature range from 625 to 780 °C is seen in NiSO_4_ system (Fig. 2c). Those effects are accompanied by the mass loss on the TG curves (1.5 and 4.5 %, respectively) probably due to the phase transformation (dehydroxylation) of mixed LDH and removing SO_4_^2−^. The insignificant part of mixed LDHs in the phase composition of the surface structures (NiCl_2_ and CuSO_4_ systems, Fig. 1b, h) correlates with a relatively small mass loss (Fig. 2d, b) by the samples (4.5 and 5.4 %, respectively).

The shape of the TG curves permits to separate the parts of adsorbed and structural water and, as a result, it becomes possible to estimate the quantitative phase distribution in every system. The full characteristic of the thermal effects is summarized in Table 4.

**Table 4:** The characteristic of thermal effects of the phases that are formed on the steel surface

The activator solution	Endoeffects, °C		Exoeffects, °C		Total mass loss, %
	Removing adsorbed water	Dehydroxylation	Magnetite→maghemite	Maghemite→hematite	
CoSO_4_	111	274	220	326	14.7
CuSO_4_	106	285	200	340	5.4
NiSO_4_	111	272	240	324	28.2
NiCl_2_	113	281	200	338	4.5

In general, to obtain higher quality LDHs, the weak-crystallized precipitates must be aged at a high temperature. As it was shown in [29], the drying of the sediments lasted 12 h at 70 °C and their calcination was carried out at 900 °C. In another work [30], calcination of the LDH-based composites was performed in the air at 700 or 900 °C for 2 h and the resulting product (spinel CoFe_2_O_4_) was then slowly cooled to a room temperature. On the contrary, the formation of mixed LDHs on the steel surface under the RCD conditions does not require increased temperatures, and it usually takes place in the temperature range from 20 to 50 °C (Fig. 1). Mixed LDHs, as an additional phase, significantly complicate the obtaining of non-stoichiometric spinel ferrite particles, and their influence may be reduced by thermal treatment of the samples or by choosing the chemical composition of water solutions when the rotation–corrosion dispergation method is applied.

**Fig. 3:**
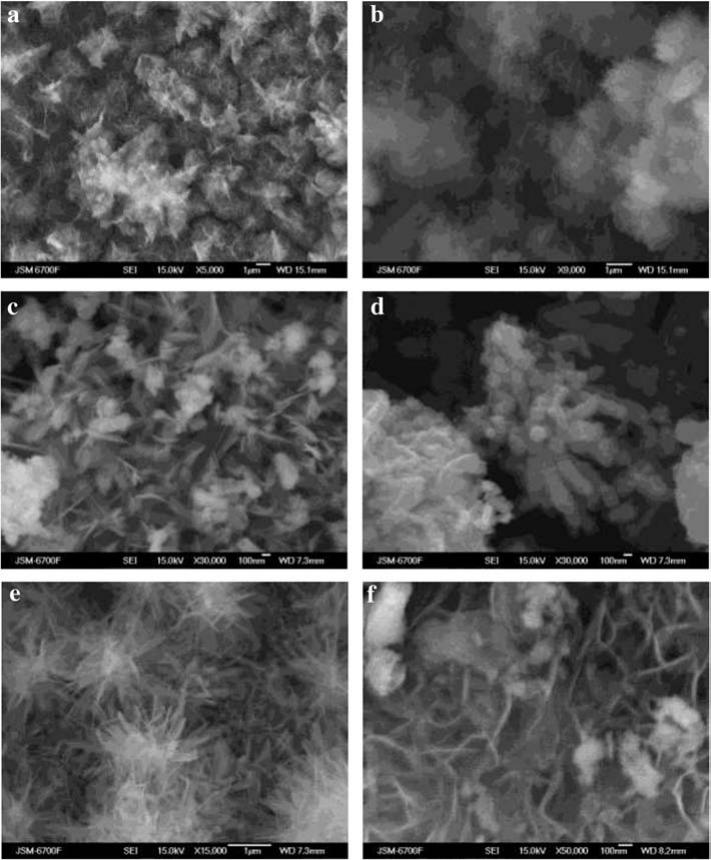
SEM images of the phases formed on the steel surface. The 3d-metal water salt solutions contacting with the steel surface: **a**, **b** CoCl_2_; **c**, **d** CoSO_4_; **e** CuSO_4_; **f** NiCl_2_

The formation of mixed LDHs and spinel ferrites is considered as two unrelated processes under the RCD conditions [31]. The solid-state transformation of LDH precursors takes place when the ‘wet chemistry’ is used to obtain the spinel ferrites. So, the inheritance of the platelet-like LDH morphology by spinel ferrites was explained by a topotactic transformation of the platelet-like LDH precursors [4]. The hierarchical morphology of the corresponding LDH precursors was confirmed by the resulting CoFe_2_O_4_ materials [30]. The typical morphology of the mineral phases formed on the steel surface when it was contacting with water inorganic salt solutions within 24 h is shown in Fig. 3. Also, Fig. 3a, b demonstrate the mixed hydroxycarbonate-hydroxychloride cobalt-bearing LDHs, whereas Fig. 3c, d show the plate-like hydroxysulfate cobalt-bearing LDHs and spherical spinel ferrite particles. The sample of the hydroxysulfate copper-bearing LDH is present in Fig. 3e. The mixed hydroxycarbonate–hydroxychloride nickel-bearing LDHs and respective spinel ferrite particles are seen in Fig. 3f.

## Conclusions

The kind of 3d-metal cations in water solutions influences the phase composition of iron–oxygen sediments, e.g., the polymorphic modification of ferric oxyhydroxides (goethite and lepidocrocite). XRFS data confirmed that 3d-metal cations (Co, Ni, Zn, and Cu) associate not only with spinel ferrite phases but with mixed layered double hydroxides as well as ferric oxyhydroxides. At that, the spinel ferrites and mixed LDHs, formed under the RCD conditions, belong to the non-stoichiometric mineral phases, and the first can be distinguished as the magnetite doped by various cations. The usage of mixed LDH precursors to obtain spinel ferrite particles includes their gradual solid-state transformation accompanied by the inheritance of the platelet-like LDH morphology by spinel ferrites. On the contrary, the application of the rotation–corrosion dispergation method leads to simultaneous re-precipitation of the plate-like mixed LDH structures and spherically shaped spinel ferrite particles. Thermal treatment of the mixed LDHs among other mineral phases formed via the RCD process permits to obtain the homogenous nanoparticles of spinel ferrites and the maghemite or hematite doped by 3d-metals.
